# Supplementary material for: The Impact of Psychological Interventions with Elements of Mindfulness (PIM) on Empathy, Well-Being, and Reduction of Burnout in Physicians: A Systematic Review
Source: Int J Environ Res Public Health. 2021 Oct 25;18(21):11181. doi: 10.3390/ijerph182111181 (PMC8582910; doi:10.3390/ijerph182111181)
Supplement: Supplementary file 1 [file ijerph-18-11181-s001.zip › ijerph-1382986-supplementary.pdf]

Supplementary Material Table S1: Characteristics of reviewed studies using physicians as study subjects

Ref No Author(s) Year	Topic/study question and study type	Sample size	Intervention/methods	Instruments/measures	Main findings	QA (MERSQI)
[33] Fortney et al (2013)	-investigating change of job satisfaction, quality of life and compassion after an abbreviated mindfulness intervention among primary care physicians<br><br>-PROSPECTIVE NON-RANDOMIZED PRE-POST INTERVENTION STUDY	N=30(2x15)<br>-primary care physicians (family medicine, internal medicine, pediatrics)<br>-United States of America	-abbreviated 18h mindfulness course<br><br>-measures at 4 points (baseline/day 1/week 8/after 9 months)	-Maslach Burnout Inventory (MBI)-job satisfaction<br>-Depression Anxiety Stress Scales 21 (DASS-21)-quality of life (QOL)<br>-Perceived Stress Scale (PSS) – QOL<br>-Resilience Scale (RS) – QOL<br>Santa Clara Briel Compassion Scale (SCBC) - compassion	-significant reduction in measures of burnout, depression, anxiety and stress on all 3 follow-up surveys<br>-MBI change (week 8 vs baseline): EE -5.5, D -2.3, PA 3.1	12
[44] Amutio et al (2014)	-the impact of a mindfulness-based stress reduction (MBSR) program on improving well-being.<br><br>-RANDOMIZED CONTROLLED TRIAL	N=42<br>-physicians<br>-Spain	-8-week MBSR program with 10-month maintenance period<br><br>-measures pre/mid/post	-Five Facets of Mindfulness Questionnaire (FFMQ-Spanish version)<br>-Smith Relaxation States Inventory (SRSI-3)<br>-Heart rate	-significant improvements in the levels of mindfulness and relaxation<br>-significant reductions in heart rate<br>-results maintained and improved 10 months after the end of intensive phase	14
[34] West et al (2014)	-influence of facilitated physician small-group curriculum on well-being<br><br>-RANDOMIZED CONTROLLED TRIAL	N=74<br>-internal medicine physicians<br>-United States of America	-19 biweekly facilitated physician discussion groups incorporating elements of mindfulness, reflection, shared experience, and small-group learning for 9 months<br><br>-measures at baseline/every 3 months during the study/3 and 12 months after	-Physician Job Satisfaction Scale<br>-Empowerment at Work Scale<br>-Quality of Life<br>-Medical Outcomes Study Short-Form Health Survey<br>-Maslach Burnout Inventory<br>-Perceived Stress Scale<br>-Positive Depression Screen<br>-Jefferson Scale of Physician Empathy	-improvement in elements of physician well-being, including meaning, empowerment, and engagement in work, and reduction of distress, including depersonalization<br>-results sustained after 12 months<br>-MBI change post intervention (month 9): overall burnout -24.7%, D -15.5%, EE -19.4%	13
[35] Pflugeisen et al (2016)	-evaluating the feasibility of implementing a video-module-based mindfulness pilot program intended to reduce stress, improve well-being, and develop mindfulness skills<br><br>-PROSPECTIVE NON-RANDOMIZED PRE-POST INTERVENTION STUDY	N=23<br>-physicians<br>-United States of America	-8 week mindfulness training (three 90-min in-person trainings, weekly online video-module trainings and weekly teleconference coaching calls)<br><br>-measures pre/week 8/week 16	-Perceived Stress Scale<br>-Maslach Burnout Inventory<br>-Kentucky Inventory of Mindfulness Skills	-significant decrease in stress and emotional exhaustion, increase in mindfulness skills and sense of personal accomplishment<br>-results sustained after 8 weeks<br>-MBI mean (SD) baseline/week 8: EE 27.3 (11.0)/22.1 (11.3), D 8.2 (6.1)/6.5 (4.1), PA 38.0 (7.1)/42.1 (7.3)	9

Ref No<br>Author(s)<br>Year	Topic/study question and study type	Sample size	Intervention/methods	Instruments/measures	Main findings	QA (MERSQI)
[45]<br>Verweij et al<br>(2016)	-feasibility and effectiveness of MBSR on burnout, empathy, and work-related wellbeing of GPs<br><br>-CONTROLLED MIXED METHODS PRE-POST INTERVENTION STUDY	N=50<br>-GPs<br>-Netherlands	-8-week MBSR<br><br>-measures pre/post	-Utrecht Burnout Scale for Contactual Occupations (UBOS-C) – validated Dutch version of MBI<br>-Utrecht Work Engagement Scale<br>-Jefferson Scale of Empathy (JSE)-student version<br>-Five Facet Mindfulness Questionnaire (FFMQ)	-MBSR for GPs is feasible and might result in fewer burnout symptoms and increased work engagement and wellbeing<br>-no increase in empathy<br>-qualitative data generated interesting themes on the range of possible effects or mediating factors of MBSR	10.5
[36]<br>Krasner et al<br>(2009)	-determining whether an intensive program is associated with improvement in well-being, psychological distress, burnout, and capacity for relating to patients<br><br>-PROSPECTIVE NON-RANDOMIZED PRE-POST INTERVENTION STUDY	N=70<br>-primary care physicians<br>-United States of America	-the course included: mindfulness meditation, self-awareness exercises, narratives about meaningful clinical experiences, appreciative interviews<br>-8-week intensive phase (2.5 h/wk, 7-hour retreat) and 10-month maintenance phase (2.5 h/mo)<br><br>-measures at baseline/at 2, 12, 15 months	-2-Factor Mindfulness Scale<br>-Maslach Burnout Inventory<br>-Jefferson Scale of Physician Empathy<br>-Physician Belief Scale<br>-Mini-markers of the Big Five Factor Structure<br>-Profile of Mood States (POMS)	-short-term and sustained improvements in well-being and attitudes associated with patient-centered care<br>-increases in mindfulness correlated with reductions in burnout and total mood disturbance<br>-improvements in patient-centered qualities were correlated with increases in mindfulness<br>-MBI mean pre/post week 8: EE 27.8/23.7, D 8.6/7.6, PA 41.2/42.0	10.5
[37]<br>Sood et al<br>(2014)	-the efficacy of SMART program for decreasing stress and anxiety and improving resilience and quality of life<br><br>-RANDOMIZED CONTROLLED PILOT STUDY	N=22<br>-physicians (Department of Radiology)<br>-United States of America	-SMART (Stress Management and Resiliency Training) program (a single 90-min group session with two follow-up phone calls)<br><br>-measures at baseline and week 12	-Perceived Stress Scale (PSS)<br>-Smith Anxiety Scale (SAS)<br>-Linear Analog Self-Assessment Scale (LASA)<br>-Mindful Attention Awareness Scale (MAAS)<br>-Connor-Davidson Resilience Scale (CD-RISC)	-statistically significant and clinically meaningful improvement in anxiety, stress, quality of life and mindful attention	11.5
[38]<br>Beckman et al<br>(2012)	-understanding what aspects of mindful communication contributed to physicians' well-being and the care they provide<br><br>-QUALITYTIVE STUDY	N=20<br>-primary care physicians<br>-United States of America	-52-hour mindful communication program (8 weekly sessions, silent retreat, 10 monthly sessions)<br><br>-in-depth semistructured interviews	/	-improvement in the participants' ability to be attentive and listen deeply to patients' concerns, respond to patients more effectively, and develop adaptive reserve<br>-sharing personal experiences from medical practice with colleagues reduced personal isolation	/

Ref No Author(s) Year	Topic/study question and study type	Sample size	Intervention/methods	Instruments/measures	Main findings	QA (MERSQI)
[47] Gill et al (2014)	-the role of discussion groups in encouraging and supporting the development of professionalism among doctors<br><br>-QUALITYTIVE STUDY	N=56<br>-physicians of various specialities<br>-United Kingdom	-workplace-based discussion groups including doctors from all non-consultant grades and specialties<br><br>-questionnaire given pre/after 6 months<br>-interviews	/	-improved understanding of professionalism at an individual level and<br>increased awareness of the collective nature of professionalism in everyday clinical practice	/
[48] Hamilton-West (2018)	-to explore whether a modified mindfulness-based cognitive therapy (MBCT) has the potential to reduce stress and burnout<br><br>-MIXED METHOD PRE-POST INTERVENTION STUDY	N=22<br>-NHS general practitioners<br>-United Kingdom	-8-week MBCT<br><br>-measures at baseline/after 1 month/3 months	-Maslach Burnout Inventory (MBI)<br>-Perceived Stress Scale (PSS)<br><br>-qualitative data on personal experiences	-very high compliance<br>-scores on validated measures of stress and burnout improved significantly at 1 month follow-up<br>-improvement maintained at 3 month follow up<br>-MBI mean pre/post(1 month): EE 29.7/18.3, D 9.23/6.67, PA 35.8/37.8	10

Supplementary Material Table S2: Results of included studies for residents/interns

No Author(s) Year	Topic/study question and study type	Sample size	Intervention/methods	Instruments/measures	Main findings	QA (MERSQI)
[32] Ireland et al (2017)	-effectiveness of a mindfulness training intervention in reducing stress and burnout among medical practitioners<br><br>-RANDOMIZED CONTROLLED TRIAL	N=44<br>-intern doctors<br>-Australia	-1:active control (1h extra break per week)<br>-2: 10-week mindfulness training<br><br>-measures pre/mid/post int.	-Copenhagen Burnout Inventory (CBI)<br>-Perceived Stress Scale (PSS)	-significant reduction in stress and burnout for group 2 (10 week mindfulness training)	14

Ref No Author(s) Year	Topic/study question and study type	Sample size	Intervention/methods	Instruments/measures	Main findings	QA (MERSQI)
[39] Taylor et al (2016)	-feasibility and impact of a brief mindfulness intervention<br><br>-PROSPECTIVE NON-RANDOMIZED PRE-POST INTERVENTION STUDY	N=33<br>-pediatric residents<br>-United States of America	-10 days program of mindfulness meditation using a free smartphone application<br><br>-measures pre/post int.	-Maslach Burnout Inventory (MBI)<br>-Mindful Attention Awareness Scale (MAAS)	-increased percentage perceived mindfulness as useful<br>-statistically significant increase in number of residents who planned to discuss mindfulness as a therapeutic option for their patients<br>-MBI scores not reported	5.5
[40] Wen et al (2017)	-effect of mindfulness and meditation practice on wellness<br><br>-PROSPECTIVE NON-RANDOMIZED PRE-POST INTERVENTION STUDY	N=43<br>-resident physicians (departments of general surgery, anaesthesia, obstetrics and gynecology)<br>-United States of America	-4 week study with use of a self-guided, smartphone-based mindfulness app Headspace<br><br>-measures pre/2 weeks/4 weeks	-Positive and Negative Affect Schedule (PANAS)-mood<br>-Freiburg Mindfulness Inventory (FMI)	-significant improvement in both positive affect and mindfulness scores<br>-no change in negative affect score	9
[49] Bu et al (2019)	-assessing feasibility of a mindfulness intervention to reduce stress and burnout<br><br>-PROSPECTIVE NON-RANDOMIZED PRE-POST INTERVENTION STUDY	N=20<br>-junior doctors<br>-United Kingdom	-6-week »Mindfulness in the Workplace« course<br><br>-measures pre/post	-self-reported stress levels (1-10)<br>-secondary measures (self-reported mindfulness, its impact on wellbeing, impact on working life, impact on relationships with patients) included in a questionnaire – no validated instruments used	-statistically significant reduction in self-reported stress	10.5
[41] Bentley (2018)	-assessing the impact of an empathy training course based in relational mindfulness on burnout and empathy<br><br>-MIXED METHOD PRE-POST INTERVENTION STUDY	N=7<br>-psychiatry residents<br>-United States of America	-8-week integrated relational mindfulness and empathy training<br><br>-measures pre/post	-Helpful Responses Questionnaire (HRQ)<br>-Maslach Burnout Inventory-Human Services Survey (MBI-HSS)<br>-Learning Experiences Questionnaire (LEQ)-developed by the authors to qualitatively evaluate the course	-significant improvement on all three burnout subscales and on the measure of empathy<br>-increased awareness of their cognitive and emotional experiences<br>-increased ability to care for themselves, patients and families<br>-MBI mean pre/post: EE 27.83/25.83, D 13.5/12.83 (non-significant changes in both), PA 38.33/36.83 (decrease)	6
[42] Zazulak et al (2017)	-the impact of a course on the empathic response of medical residents<br><br>-PROSPECTIVE NON-RANDOMIZED PRE-POST INTERVENTION STUDY	N=15<br>-resident trainees (obstetrics and gynaecology and family medicine)<br>- United States of America	-»Art of Seeing« course including arts-based visual literacy and mindfulness practice<br><br>-measures pre/post	-Interpersonal Reactivity Index (IRI)<br>-Compassion Scale<br>-Five Facet Mindfulness Scale<br><br>-semistructured interviews	-improvement in the Mindfulness Scale domains related to self-confidence and communication<br>**-majority of psychometric measures did not reveal differences between groups**<br>-positive impact on the perceived empathy towards colleagues and patients, and on the perception of personal and professional well-being	10

Ref No<br>Author(s)<br>Year	Topic/study question and study type	Sample size	Intervention/methods	Instruments/measures	Main findings	QA (MERSQI)
[46]<br>Verweij et al<br>(2017)	-effectiveness of MBSR in reducing burnout<br><br>-PROSPECTIVE NON-RANDOMIZED PRE-POST INTERVENTION STUDY	N=148<br>-residents from medical, surgical and primary care disciplines<br>-Netherlands	-MBSR (8 weekly 2.5h sessions and one 6h silent day)<br><br>-measures pre/after 3 months	-Utrecht Burnout Scale (UBOS-C)<br>-Penn State Worry Questionnaire<br>-Work-home Interaction NjimeGen<br>-Five-Facet Mindfulness Questionnaire Short Form<br>-Self-Compassion Scale Short Form<br>-Mental Health Continuum-Short Form<br>-Jefferson Scale of Physician Empathy	**-no significant difference in emotional exhaustion**<br>-significant improvements in personal accomplishment, mindfulness skills, self-compassion and perspective-taking	**13.5**
[43]<br>Runyan et al<br>(2016)	-to evaluate the impact of a new 1-month wellness curriculum on burnout, empathy, stress and self-compassion<br><br>-PROSPECTIVE NON-RANDOMIZED PRE-POST CONTROLLED INTERVENTION STUDY	N=12<br>-family medicine residents<br>-United States of America	-1-month wellness curriculum<br><br>-measures pre/3-month follow-up	-Maslach Burnout Inventory (MBI)<br>-Self Compassion Inventory (SCI)<br>-Perceived Stress Scale (PSS)<br>-Jefferson Empathy Scale (JES)	-positive trends of all the measures, particularly the mindfulness scale of the SCI and the JES<br>-MBI mean pre/3 month post: EE 20.44(9.36)/18.00(9.88), cynicism 15.67(8.94)/15.33(8.07), professional efficacy 24.78(7.68)/26.89(4.29)	9
